# Comparative analysis of phytonutrients of *Moringa oleifera* leaves from South Africa and Nigeria, and their antimicrobial and antioxidant potentials by UPLC-ESI-QToF-MS and OPLS-DA chemometric analysis

**DOI:** 10.3389/fnut.2024.1490484

**Published:** 2025-01-27

**Authors:** Jonathan Kayembe, Cosa Sekelwa, Kokoette Bassey

**Affiliations:** ^1^Department of Pharmaceutical Sciences, School of Pharmacy, Sefako Makgatho Health Sciences University, Ga-Rankuwa, South Africa; ^2^Department of Biochemistry, Genetics and Microbiology, University of Pretoria, Hatfield, South Africa

**Keywords:** phytonutrients, chemical markers, chemometrics, *Moringa oleifera* leaves, comparative, comparative analysis

## Abstract

**Background:**

*Moringa oleifera* Lam. has bioactive phytonutrients in abundance and offers diverse health benefits. The leaves of this plant have established significance in traditional medicine and nutrition. It is traditionally used by Nigerian and South African mothers to mitigate undernutrition. Usually, the powder leaves are added to porridge to feed the children. This study aimed to conduct a comparative analysis of the phytonutrients (nutrients protectors) or supplements, antioxidant, and antimicrobial potentials of *M. oleifera* leaves from Nigeria and South Africa to benchmark quality control protocols for commercial beverages such as *Moringa* porridge.

**Methods:**

Standard techniques, including high-performance liquid chromatography-photodiode array and ultra-high-performance liquid chromatography electrospray ionization quadruple time-of-flight mass spectrometry (UPLC-ESI-QToF-MS) and chemometrics orthogonal partial least square discriminant analysis (OPLS-DA) were employed for phytoconstituents fingerprinting. Whereas the antioxidant potentials of the extracts were determined using 2,2-diphenyl-1-picrylhydrazyl (DPPH) and hydrogen peroxide scavenging assays, the antimicrobial potentials of the extracts were evaluated using minimum inhibitory concentrations protocol.

**Results:**

The chemometric analysis with a line regression (R2) = 0.97 revealed 70% significant similarities in the phytonutrients of samples between the two regions and an intriguing 30% variation within the same plant species. In addition, kaempferol, quercetin, luteolin, tangutorid E, and podophyllotoxin, among others were annotated as the major phytonutrients in the samples. The antioxidant assays unveiled concentration-dependent trends with scavenging activity of up to 98% (half-maximal inhibitory concentration [IC_50_] = 0.14 mg/mL) for 2,2-diphenyl-1-picrylhydrazyl (DPPH) and 87% (IC_50_ = 0.28 mg/mL) for hydrogen peroxide assay. All the test extracts did not exhibit good to significant antibacterial inhibitory effect (minimum inhibitory concentration [MIC] = 1.25 mg/mL) compared to ciprofloxacin (MIC = 0.0156–0.0039 mg/mL).

**Conclusion:**

The variations in the phytonutrients of the same *M. oleifera* species harvested from different countries could have dire consequences including potential health risks and even death. This study should serve as a benchmark toward the phytonutrients and marketing implications on the quality of products formulated with samples harvested from different growth environments and exists as a reference for further research into the cultivation and marketing of *M. oleifera* leaves in South Africa.

## 1 Introduction

### 1.1 Background

*Moringa oleifera* Lam., also known as the Horseradish tree, is a plant with a long history of use in traditional medicine and as a source of nutrition (1). It is native to the Indian subcontinent but is grown in many parts of the world, including South Africa and Nigeria (2, 3). The leaves of the *M. oleifera* tree are particularly rich in phytonutrients, including phenolics, flavonoids, and alkaloids, which are also known as nutrient protectors. These phytonutrients have demonstrated a wide range of biological activities, including antioxidant, anti-inflammatory, and antimicrobial properties (4). The use of *M. oleifera* leaves as a natural remedy for various diseases has been documented for centuries, and recent scientific studies have validated many of the traditional uses. For example, *M. oleifera* leaves have been shown to have hypoglycemic, hypolipidemic, and anti-inflammatory effects, among others (5). The high nutritional value of *M. oleifera* leaves has also been well-documented, with elevated levels of protein, vitamins, and minerals; therefore, *M. oleifera* leaves are used to combat undernutrition in children (5–8). While there have been some studies on the phytonutrient’s composition of *M. oleifera* leaves from different regions (9), there is still a clear need for more comparative studies which can determine if there are any significant differences in the phytonutrients composition of *M. oleifera* leaves from different regions. This information could have important implications for the use of *M. oleifera* leaves as a source of natural phytonutrients for medicinal and nutritional purposes (2, 7, 9).

*M. oleifera* is a versatile plant widely distributed across the globe, including tropical and subtropical regions of Africa, Asia, and South America. The environmental conditions of the regions in which *M. oleifera* grows can significantly affect its growth, physiology, and phytonutrients composition (3, 10, 11). For example, climatic conditions, such as temperature, rainfall, and humidity, can influence the growth rate and the accumulation of bioactive phytonutrients in *M. oleifera*. Additionally, soil type, nutrient availability, and altitude can also impact the chemical composition of the plant (11). Worldwide, the interest and demand for herbal medicines have increased recently. Due to this, the production and marketing of herbal remedies have attracted more attention including regulation of their sales. However, some products still slip through the cracks because of the lack of scientific research on these products. For example, beverages, such as Moringa tea, Moringa porridge, Alomo bitters, Coco Samba, Jekonmo, Odogwu Bitters, Olekoko Energy Bitters, among many other herbal concoctions, are used as sex activity boosters and as on-the-go energy drinks (12, 13) that are traded from Nigeria to South Africa. The requirement for scientific validation and standardization of herbal medicines is a significant obstacle. To meet the rising demand for natural remedies, both nations are working to create high-quality herbal medicines that adhere to international standards. The bilateral trade relationship between both countries calls for validating some plant species from both countries to avoid product adulteration and the health consequences that come with that.

In this study, we aimed to compare the phytonutrients of *M. oleifera* leaves from South Africa and Nigeria using high-performance liquid chromatography with photodiode array detection (HPLC-PDA) and ultra-high performance liquid chromatography electrospray ionization-quadruple time-of-flight mass spectrometry (UPLC-ESI-QToF-MS) for the analysis of leaf samples from both countries. HPLC-PDA was used to generate multivariate data that was made compatible with chemometrics orthogonal partial least squares discriminant analysis (OPLS-DA) using MetaboAnalyst—a free web-based software. Samples nominated by the software as those responsible for recognized patterns and clustering were further analyzed using high-resolution UPLC-ESI-QToF-MS to annotate the names and the structures of phytonutrients responsible for the clustering. This study’s results could help in establishing a baseline for the phytonutrient composition of *M. oleifera* leaves from different regions and could inform the development of new natural remedies and nutritional supplements. Additionally, understanding the composition of phytonutrients of *M. oleifera* leaves from different regions could help in identifying the most potent sources of bioactive phytonutrients and inform strategies for the sustainable cultivation of *M. oleifera*.

Due to the wide use of *M. oleifera* leaves to mitigate malnutrition—a function of their rich phytonutrient content—this study also evaluated its antioxidant and antibacterial potentials. This exploration was fueled by the urgency to address the impending antimicrobial resistance and the growing need for natural management of oxidative stress and related diseases.

## 2 Materials and methods

### 2.1 Plant material collection and comminution

Fresh leaves of *M. oleifera* from Nigeria were identified by a botanist from the Department of Pharmacognosy, Faculty of Pharmacy, Obafemi Awolowo University (OAU), Ile-Ife, Osun State Nigeria (7_31014.761200 N, 4_31049.134000 E). The leaf samples were harvested from (*n =* 35) trees; the leaves were collected, sorted, and processed, and voucher specimen NGMOL01–NGMOL035 was allocated to the respective sample. From a different set *n =* 35 trees, more leaf samples from South Africa were collected from Botanica Natural Products—a medicinal plants farm located in Limpopo Province (−22°39′59.99 S, 29^°^05′60.00 E), the samples were identified by an indigenous knowledge system (IKS) practitioner (traditional healer). Permission for the sample collection was granted by the research ethics committee of Sefako Makgatho Health Sciences University Ga-Rankuwa, South Africa (SMUREC/P/133/2020:PG). After collection, the randomly selected samples were identified by the South African Biodiversity Institute (SANBI), and voucher specimen SAMOL1–SAMOL35 were allocated to each sample. The voucher specimen NGMOL01–NGMOL035 and SAMOL1–SAMOL35 were deposited in the Department of Pharmaceutical Sciences, Sefako Makgatho Health Sciences University, Ga-Rankuwa, South Africa. A total of *n =* 70 samples were used for the study. The samples were shade-dried, ground as a fine powder, and stored at room temperature until analysis and retention samples of each sample were kept in the Department of Pharmaceutical Sciences, Sefako Makgatho Health Sciences University, Ga-Rankuwa, South Africa.

### 2.2 Chemicals and reagents

Methanol (HPLC grade) and dichloromethane (DCM) were obtained from Merck (Johannesburg, Gauteng, South Africa). Ultrapure water was obtained from a Millipore water purification system (Hach South Africa (Pty) Ltd Johannesburg, Gauteng, South Africa). HPLC grade acetonitrile was obtained from Sigma–Aldrich (Johannesburg, Gauteng, South Africa).

### 2.3 Extraction of plant material

Approximately 0.5 g of *n* = 35 Nigerian and *n* = 35 South African powdered plant materials of *M. oleifera* were extracted separately in a sonication bath for 30 min with 10 mL of methanol to afford South African/Nigerian methanol (SAM/NGM) extracts, 10-ml DCM to obtained South African/Nigerian dichloromethane (SAD/NGD) extracts, and 10-ml of 1:1 v/v mixture of methanol and DCM to yield South African/Nigerian dichloromethane:methanol (SADM/NGDM) extracts. The extracts were filtered through a 0.22-μm syringe filter and stored in a refrigerator at 4°C until analysis.

### 2.4 Chromatographic analysis

#### 2.4.1 HPLC-PDA analysis

HPLC-PDA analysis was conducted using a Shimadzu HPLC system equipped with a PDA detector and a Phenomenex Luna C18 column (411 Madrid Avenue, Torrance, CA 90501–1430 USA). (250 mm × 4.6 mm, 5 μm). The mobile phase consisted of 0.1% formic acid in water (solvent A) and 0.1% formic acid in acetonitrile (solvent B), and the flow rate was set at 1 mL/min. The gradient elution process was set as follows: 0–2 min, 5% solvent B; 2–10 min, 5–20% solvent B; 10–20 min, 20–35% solvent B; 20–30 min, 35–60% solvent B; 30–40 min, 60–70% solvent B; 40–50 min, 70–95% solvent B; 50–60 min, and 95–5% solvent B. The column temperature was maintained at 30°C, and the injection volume was 10 μL.

#### 2.4.2 UPLC-ESI-QToF-MS analysis

The UPLC-ESI-QToF-MS analysis was conducted by introducing 1.0 mg/mL of each test extract into a UPLC (Waters Acquity UPLC; Waters, Milford, MA, United States). The quantitative data-independent acquisition (DIA) was carried out using two simultaneous acquisition functions with low- and high-collision energy (MSe collision approach) with a QToF instrument. The high-energy MS scan was time-aligned with the low-energy scan to predict which fragment ions belong to which precursor ions; consequently, the full mass spectrum was acquired. Fragmentation patterns were used for qualitative confirmation. Fragmentation was performed using high-energy collision-induced dissociation (CID). The fragmentation energy was set at 2 and 3 V for the trap and collision energy, respectively. The ramping was set from 3–4 to 20–40 V for the trap and transfer collision energy, respectively. Mass spectral scans were collected every 0.3 s. The raw data were collected in the form of a continuous profile. Mass-to-charge ratios (*m*/*z*) between 50 and 1,200 Da were recorded. The test extracts were separated on a Kinetex^®^ 1.7-μm EVO C18 100 Å (2.1 mm ID × 100 mm length; Waters) column temperature, maintained at 50°C and autosampler temperature of 3°C with an injection volume of 5 μL and a flow rate 0.3 mL/min. The mobile phase consisted of 0.1% aqueous formic acid (solvent A) and HPLC-grade (Merck^TM^, Johannesburg, Gauteng, South Africa) acetonitrile 0.1%/formic acid (solvent B) at a flow rate of 0.3 mL/min. Gradient elution was applied as follows: 97% solvent A:3% solvent B for 10 s, then 100% solvent B in 16 min, changed to 97% solvent A: 3% solvent B in 50 s (held for 3.5 min), in a total run time of 20 min. Data were managed by MassLynx™ (version 4.1 UNIFI) software. The UPLC system was interfaced with a combination time-of-flight/quadrupole Xevo G2QT mass spectrometer (Waters, United States) with sampling and extraction cones set at 20 and 4.0 V, respectively. For the UPLC–MS analyses, the same column, elution gradient and flow rate were used as before. Although both positive and negative ion modes were applied in preliminary method development, higher sensitivities, and more information were obtained in the positive mode. The mass spectrometer was therefore operated in positive ion electrospray mode using nitrogen as the desolvation gas at a flow rate of 600 L/Hr. A desolvation temperature of 300°C and a source temperature of 120°C were used. The capillary and cone voltages were set to 2.6 kV (ESI+) and 2.4 kV (ESI−), respectively. Data were collected in the range *m*/*z* 50–1,200.

### 2.5 Chemometric analysis using MetaboAnalyst

The raw HPLC-PDA data were processed using Microsoft Excel^®^ 2016 speadsheet and made compatible for analysis using MetaboAnalyst 5.0—a free web-based software (14). This software is suitable for high-throughput multivariate data analysis. MetaboAnalyst 5.0 (Wishart Research Group, University of Alberta, Canada); in this study, *n* = 70 (15) and the density plots were based on all samples using row-wise normalization—normalization to sample median; data transformation; log10 normalization; and pareto scaling to afford the best model, which were indicated by the statistical results. Chemometric analysis of the HPLC-PDA data was carried out following an untargeted approach using hierarchical cluster analysis (HCA) on principal components analysis (PCA). However, the best results were obtained when OPLS-DA models were constructed and used to analyze the pretreated HPLC-PDA data. To identify the marker variables, the loadings plot, among other plot types, is usually convenient. This is because it can detect the variables responsible for cluster formation of a given dataset in addition to providing a numerical value that reflects how each original variable contributes to the plot (16). The nominated samples were further analyzed using UPLC-ESI-QToF-MS and extrapolated to identify marker phytonutrients in such samples.

### 2.6 Structural annotation of marker phytonutrients from Nigerian and South African *Moringa oleifera* leaf methanol extracts

A variable importance in projection (VIP) plot was constructed to identify the marker samples. The discrimination of the Nigerian and South African samples into two clusters depends on the variable concentrations of their phytonutrients, which the VIP plot measured in a descending order of importance. The high value of the VIP score indicates a high concentration of the phytonutrients and a corresponding outstanding contribution toward discrimination and vice versa (17). This plot was carried out using methanol, dichloromethane, and methanol-dichloromethane extracts. From the VIP plot, samples with high and low VIP values were chosen as having the highest and lowest concentrations of phytonutrients, respectively (18). The annotation of peaks in the study as well as the phytonutrients were performed by comparing the fragmentation patterns, we obtained with those available on the original Mass bank (MB) spectral data, general literature reports on *M. oleifera leaves* (19) as well as those in the extensive profiling of *M. oleifera* leaves from India and China (20). The fragmentation pattern comparison were performed by hand through physical alignment.

### 2.7 Antimicrobial activity evaluations

The method chosen to evaluate antimicrobial activity was the MIC method—a widely accepted technique for assessing the antimicrobial activities of substances, including plant extracts (21). The MIC determines the lowest concentration of a substance that effectively inhibits the visible growth of microorganisms, typically bacteria or fungi. The plant extracts were selected based on biological markers obtained from chemometric OPLS-DA analysis.

#### 2.7.1 Test pathogens and plant extracts

*Pseudomonas aeruginosa* ATCC 9721, *Streptococcus pyogenes* ATCC 19615, *Staphylococcus aureus* ATCC 25923, *Bacillus cereus* ATCC 14579, and *Escherichia coli* ATCC 10536 were selected for this study. Plant extracts: SAM31, SAM32, NGM31, NGM32, SAD31, SAD32, NGD31, NGD32, SADM31, SADM32, NGDM31, and NGDM32 (where SAM/NGM is South African/Nigerian methanol, SAD/NGD is South African/Nigerian dichloromethane, SADM/NGDM is South African/Nigerian dichloromethane: methanol 1:1 v/v extracts). Briefly, a 6 mg/mL stock concentration of different plant extracts was prepared using distilled water and mixed using a sonication bath. Resultant mixtures were tested against the aforementioned pathogens. 1% DMSO and 1 mg/mL of ciprofloxacin were prepared as negative and positive controls, respectively. The MIC was determined with the aid of a microdilution assay where 100 μL of LB broth containing each respective pathogen with the absorbance maintained at 0.08 and 0.1 was added to each well, 100 μL of the plant extract solution was also added along with 100 μL positive and negative controls which were added to additional wells. A serial dilution was performed till the last row of the plate; the plate was then incubated at 37°C for 24 h. After the 24-h period, 20 μL of iodonitrotetrazolium (INT) was added to each plate; thereafter, further incubation was performed for an hour. The MIC for each sample and product was determined by visually inspecting the wells. Red color indicated no antimicrobial activity, while bright color indicated that there was antimicrobial activity. The MIC is the lowest concentration of the extract solution at which no visible growth of the microorganism was observed. The MIC evaluations were conducted in triplicates on two separate occasions.

### 2.8 *In vitro* free radical scavenging evaluations of the *Moringa* extracts

#### 2.8.1 DPPH free radical scavenging activity of the *Moringa* extracts

This was achieved following a method previously described (22). Various concentrations ranging from 0.2 mg/mL to 1.0 mg/mL were prepared for polar methanol, non-polar dichloromethane, and methanol-dichloromethane (1:1 v/v) extracts. A DPPH solution was also created with a concentration of 0.2 mg/mL. To test the extracts’ antioxidant activity, 1.0 mL of the DPPH solution was mixed with 1.0 mL of the extract solution in a test tube and the mixture was thoroughly vortexed and then placed in a dark laboratory cupboard for 30 min to initiate the free radical scavenging reaction. The spectrophotometric absorbance of the different concentrations was measured at 517 nm using a 96-well microplate-reader spectrophotometer (SprectraMax^®^, Molecular Devices, CA, United States). Gallic acid and butylated hydroxyl toluene (BHT) were used as reference positive control at the same concentration. The percentage of radical scavenging activity of the extracts was calculated using Equation (1):


(1) $$\%\mathrm{DPPH radical scavenging activity}=A_{0}-A_{s}/A_{0}\times 100$$

where *A*_0_ is the absorbance of the negative control and *A*_s_ is the absorbance of the extracts and standards.

#### 2.8.2 Hydrogen peroxide radical scavenging activity

The hydrogen peroxide scavenging potential of the three test extracts was assessed using the method described by Hlophe and Bassey (23). For each extract, a 2-mL of hydrogen peroxide (20 mM) was prepared in a phosphate buffer saline with a pH of 7.40. To this solution, varying concentrations (ranging from 0.2 mg/mL to 1.0 mg/mL) of extract stock solutions were added in increments of 1.0 mL. The resulting mixture was vortex and incubated for 10 min before measuring the absorbance at 560 nm using the spectrophotometer discussed in subsection 2.8.1. To benchmark the anticipated results, the reference standards used for this experiment were 1.0 mg/mL of both gallic acid and butylated hydroxyl toluene (BHT) solutions. The percentage of hydrogen peroxide radical scavenging activity was calculated according to the following formula in Equation 2:


(2) %Hydroxyl peroxideradical scavenging activity=A0−As/A0×100

where *A*_0_ is the absorbance of the negative control, and *A*_s_ is the absorbance of the extracts/standards.

### 2.9 Statistical analysis

The average IC_50_ values of the replicated data obtained from the DPPH and H_2_O_2_ peroxide radical scavenging antioxidant assays were subjected to a single analysis of variance (ANOVA) test to evaluate the variance between the DPPH and H_2_O_2_ antioxidant assays.

## 3 Results

### 3.1 OPLS-DA analysis of HPLC-PDA data of Nigerian and South African *Moringa oleifera* extracts

**Figure 1:**
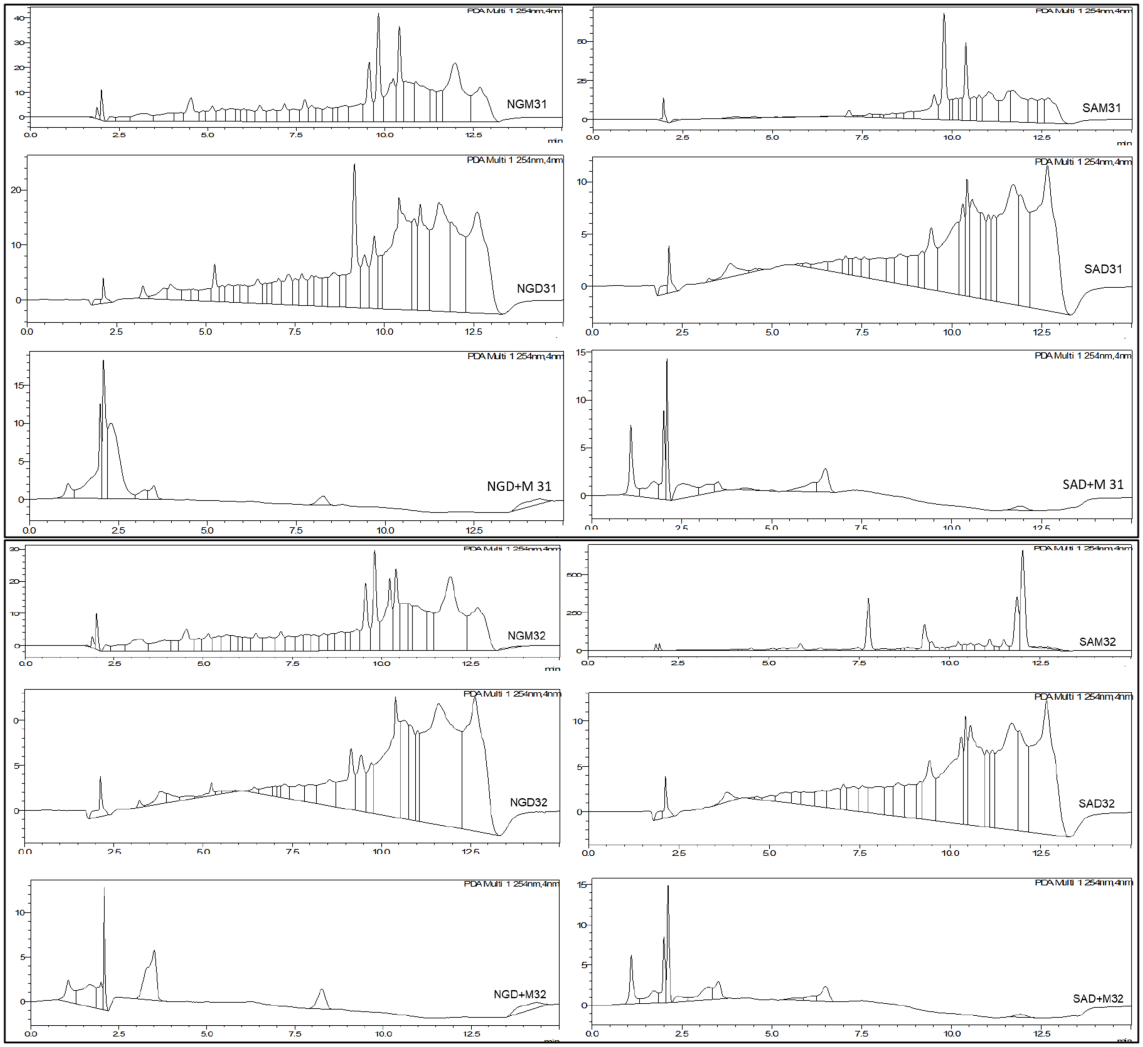
HPLC chromatograms for the selected Nigerian and South African *Moringa oleifera* leaf extracts indicating peaks that were integrated to afford area under the curve (AUC) vs. Rt.

**Figure 2:**
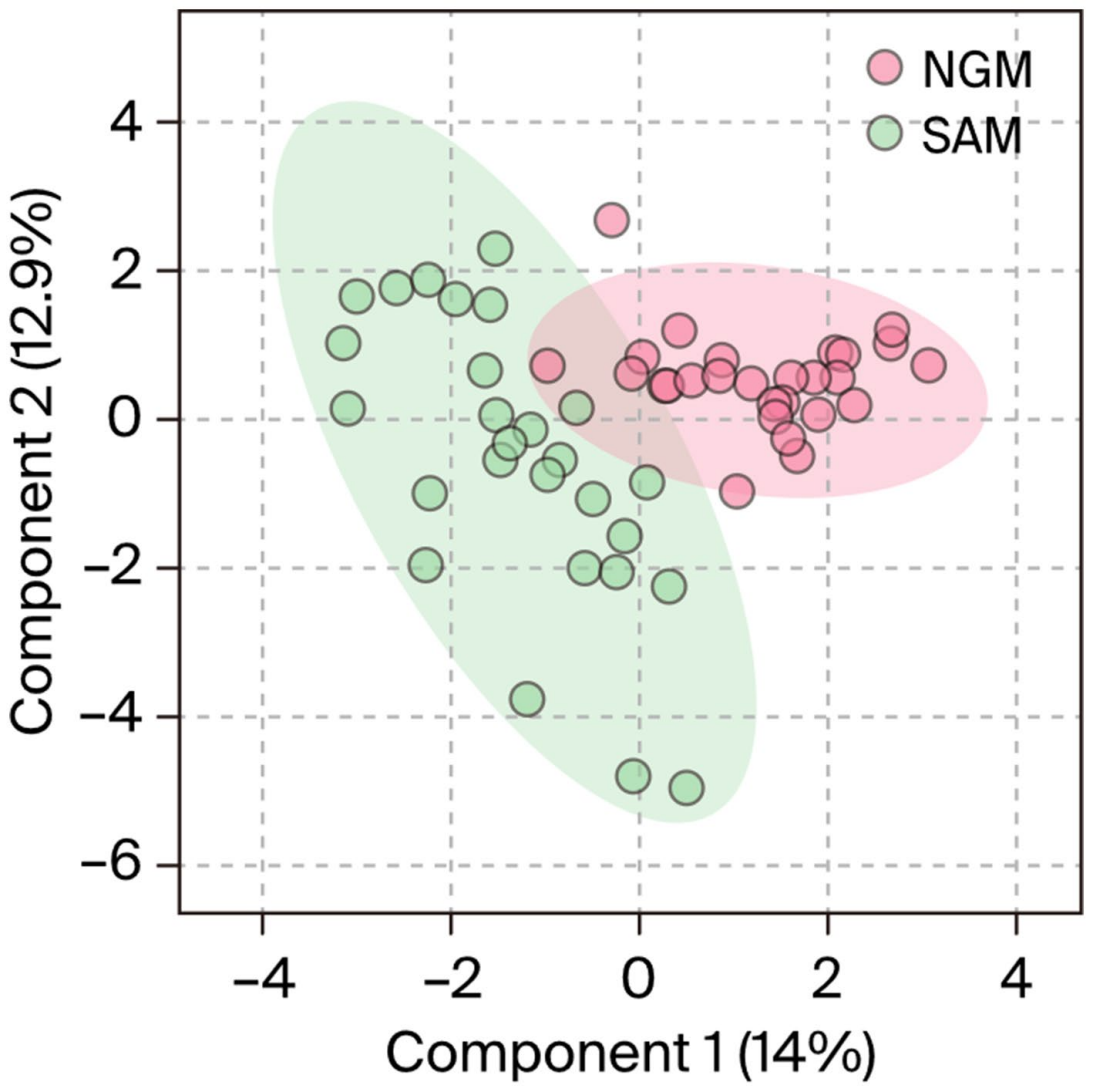
OPLS-DA analysis of Nigerian and South African *Moringa oleifera* leaf methanol extracts indicating two distinct clusters as a function of similarities–discrepancies in the phytonutrients of the samples. NGM = Nigerian methanol, SAM = South African methanol extracts.

**Figure 3:**
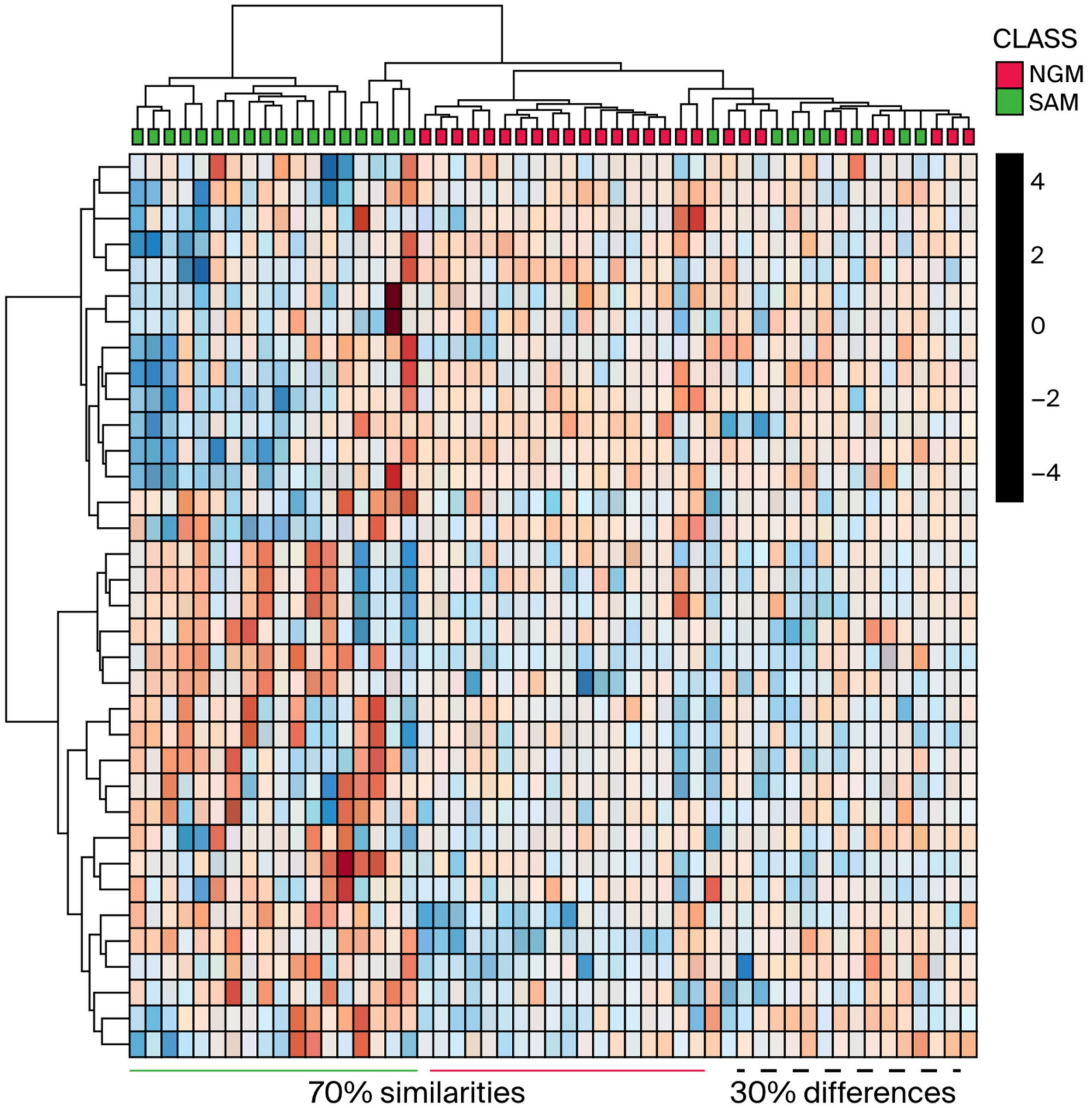
HCA plot of the Nigerian and South African methanol extract indicating the differences and similarities between the phytochemicals. NGM = Nigerian methanol, SAM = South African methanol extracts.

A total of *n* = 70 (35 Nigerian and 35 South African) samples were analyzed. These samples were extracted using methanol, dichloromethane, and methanol:dichloromethane (1:1 v/v), respectively. The HPLC-PDA chromatographic data for randomly selected samples are displayed in Figure 1. All peaks except the solvents were automatically integrated to afford the area under the curve (AUC) against the respective retention time (Rt). The AUC vs. Rt data were exported to the Microsoft Excel^®^ 2016 spreadsheet. The dataset was compatible with chemometrics analysis using MetaboAnalyst^®^ by computing the retention time in the vertical column and the area under the curve in the horizontal rows in a 37 × 41 matrix for the methanol extract. Similar prechemometrics computation of the HPLC data of the dichloromethane and methanol:dichloromethane (1:1 v/v) appeared as 37 × 49 and 37 × 21 matrices, respectively. The HPLC-chemometrics compatible data was then exported to metaboAnalyst—a free web-based software for analysis. The software recommends mean data filtering for small datasets (*n =* 70) (15). However, the data was row-wise normalized to a constant sum, log10 transformed, and finally, Pareto Scaled for better outcomes before OPLS-DA plots (Figures 1–3) were executed.

Supervised orthogonal partial least squares discriminant analysis (OPLS-DA) model with a line regression (R^2^) = 0.97 and a model prediction value of Q2 = 0.65, discriminated the dataset into two different clusters in the score plot (Figure 2). Observations of significance that influenced the sample clustering were identified for the methanol extracts. Whereas the Nigerian samples were placed in the positive cluster or quadrant (Burgundy), the South African samples were placed in the negative cluster (green). The plot also revealed 14% intergeographic location variations in the phytonutrients between the South African and Nigerian samples along the first component (t1) or *x*-axis and 12.5% intrageographic location variations between the Nigerian samples along the second component (t2) or *y*-axis. Regarding the intravariability in the NGM samples, a PC3 of the model indicated a 7.7% variation in their phytonutrients. However, there were no significant intrageographic location variations amongst the South African samples along the t2.

Even though there is a clear distinction between the *M. oleifera* leaf samples from the two countries, it is logical to envision that samples located in the plot’s mid-point may display similarities in the phytochemicals or secondary metabolite content. To visualize the possibility of similarities in the phytonutrients of Nigerian and South African *M. oleifera* leaf samples, a HCA (Figure 3) plot of the methanol extracts was conducted.

**Figure 4:**
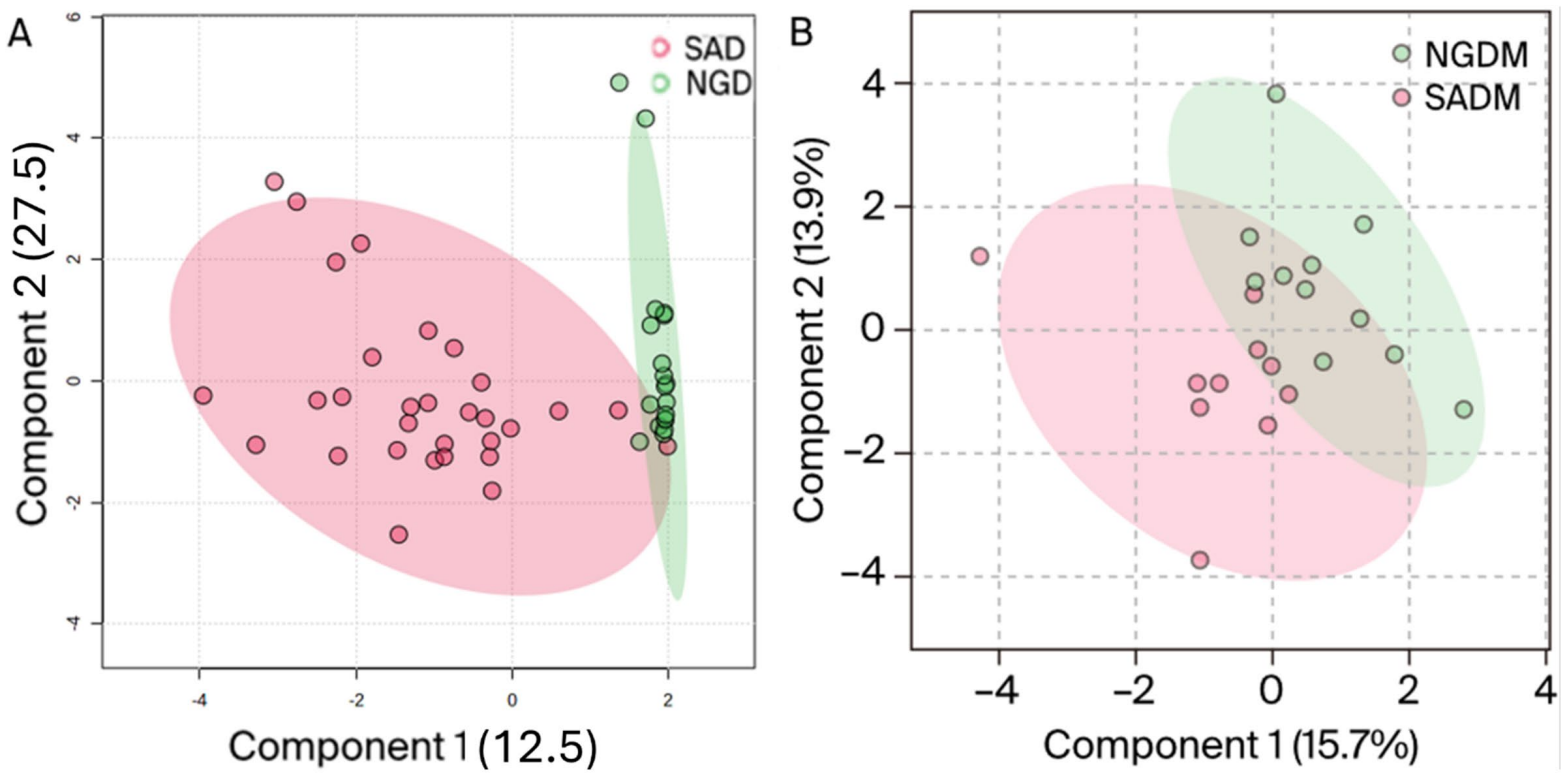
OPLS-DA score plot of Nigerian dichloromethane (NGD), South African dichloromethane (SAD) extracts **(A)**, and methanol-dichloromethane (NGDM/SADM) 1:1 v/v **(B)** portraying the variations in the phytonutrients for *Moringa oleifera* leaf samples from Nigeria and South Africa.

As shown in Figure 3, one can see that some of the samples exhibited marked dissimilarities from the left. In contrast, others displayed similarities toward the right as indicated by the green/burgundy alternating positioning of the squares in the plot. As for the *M. oleifera* leaves dichloromethane extracts, the OPLS-DA score plots afforded comparable results to those obtained from the methanol extracts. That is, the model grouped all the samples into two distinct clusters, Figure 4A. However, unlike the methanol extracts, the Nigerian DCM samples were placed out of the 95% confidence interval of the analysis (Figure 4A). On the contrary, all the South African samples, except for two outliers, were placed within the 95% confidence level. The plot also revealed 12.5% inter-geographic or class variations along the first component (t1) and 27.5% intrageographic or class variations in the secondary phytonutrients content of all the samples along the second component (t2). However, whereas comparable phytoconstituents variation results of 15.7% were obtained from the dichloromethane-methanol (1:1 v/v) extracts along component 1 (t1), a variation of 13.9% (component 2) was obtained for all the samples (Figure 4B).

### 3.2 Marker samples nomination from Nigerian and South African *Moringa oleifera* extracts by OPLS-DA analysis of HPLC-PDA data

**Figure 5:**
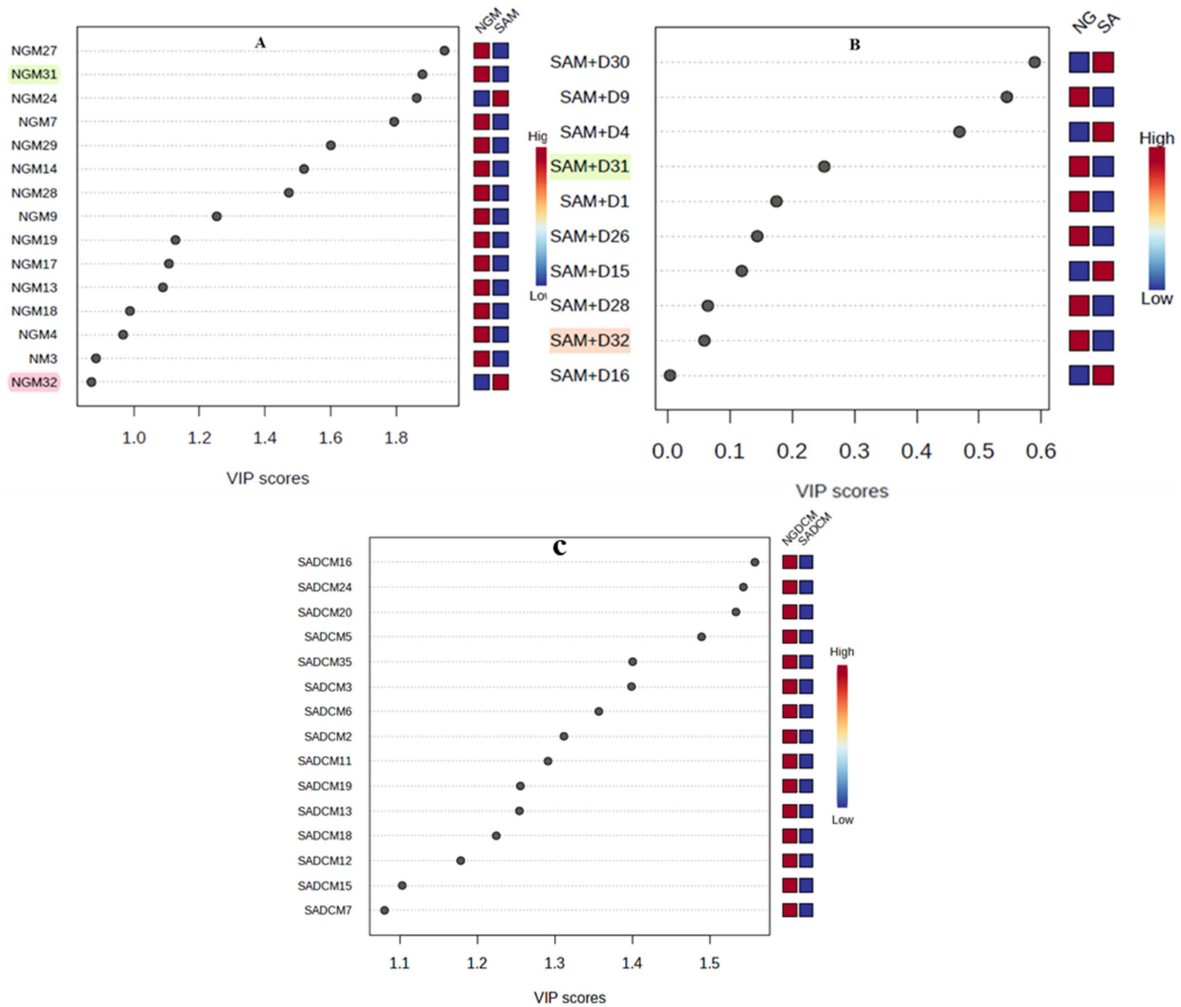
Variable importance in projection (VIP) plot showing the variability in the concentration of the phytonutrients in descending order of importance. The red and blue boxes on the right of each plot indicate whether the phytonutrient concentration is low (blue) or high (red) in the Nigerian vs. South African samples. Variations in methanol extracts **(A)**, methanol-dichloromethane (1:1 v/v) extracts **(B)**, and dichloromethane extracts **(C)**.

The marker samples often determine the clustering pattern of samples that we report in this study and the phytonutrients such samples contain. The results obtained nominated 15 samples (black dots, Figure 5) with high to low phytonutrients from each of the methanol and dichloromethane extracts (Figures 5A,C), and 10 samples from methanol-dichloromethane (1:1 v/v) extracts (Figure 5B) as the relative contributors capable of determining the phytonutrients concentration variations in the *M. oleifera* leaf samples analyzed. However, samples 31 and 32 were nominated from the methanol and methanol-dichloromethane (1:1 v/v) samples but not from the dichloromethane extract as having the highest and lowest phytonutrients, respectively. The two samples contributed between a high of 0.26–1.85 (sample 31) and a low of 0.02–0.08 (sample 32) VIP scores. Consequently, these two samples were chosen for further analysis using UPLC-ESI-QToF-MS to unravel the type of phytonutrients they contain.

### 3.3 Marker phytonutrients annotation from Nigerian and South African *Moringa oleifera* extracts by UPLC-ESI-QToF-MS profiles

**Figure 6:**
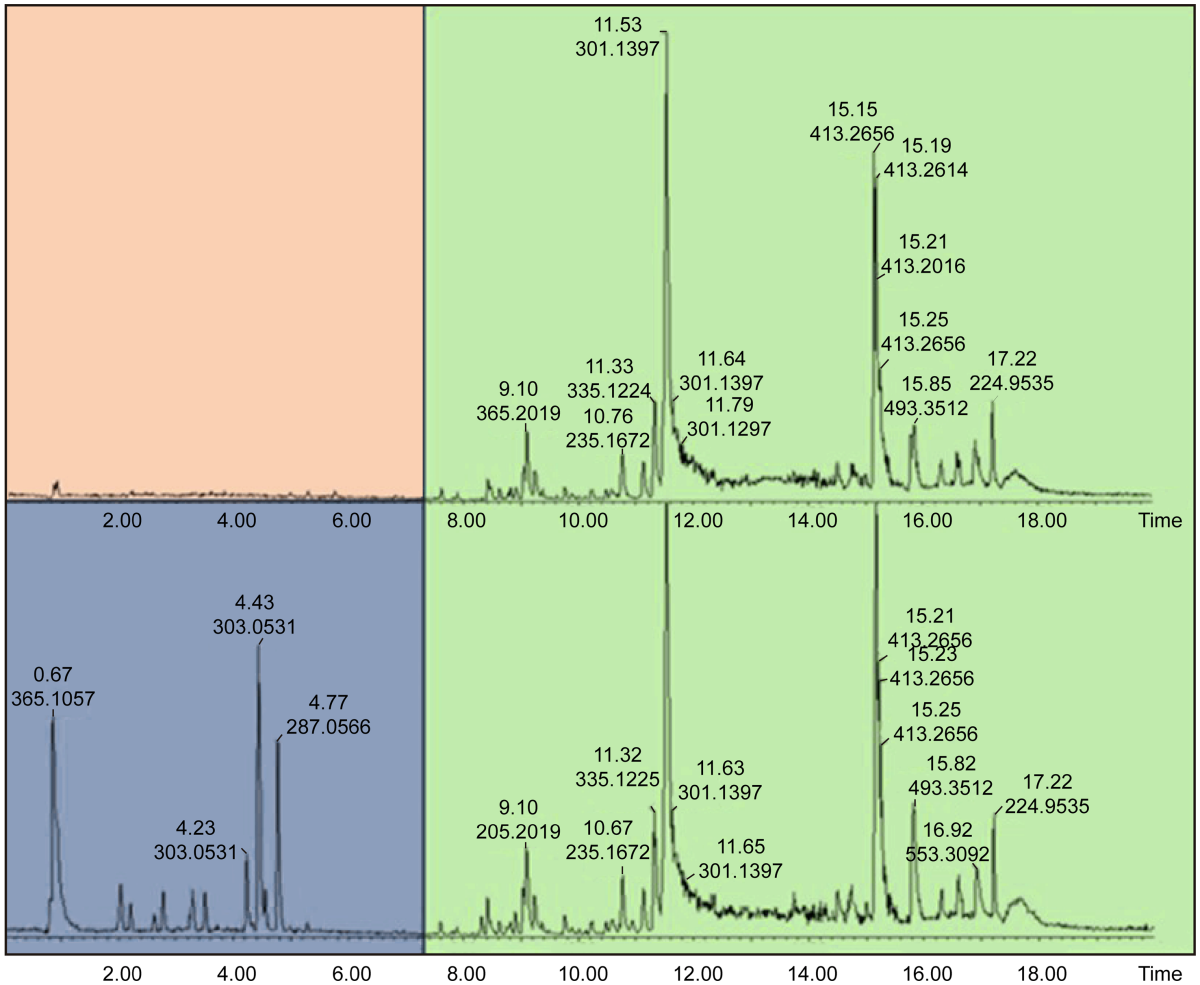
Total Ion Chromatogram (TIC) obtained UPLC-ESI-QToF-MS indicating phytochemical variations at Rt of 0.92–8.20 min and similarities at 8.25–18.00 min in NG (top) and SA (down) *Moringa oleifera* MeOH extracts. NG = Nigerian; SA = South African.

**Figure 7:**
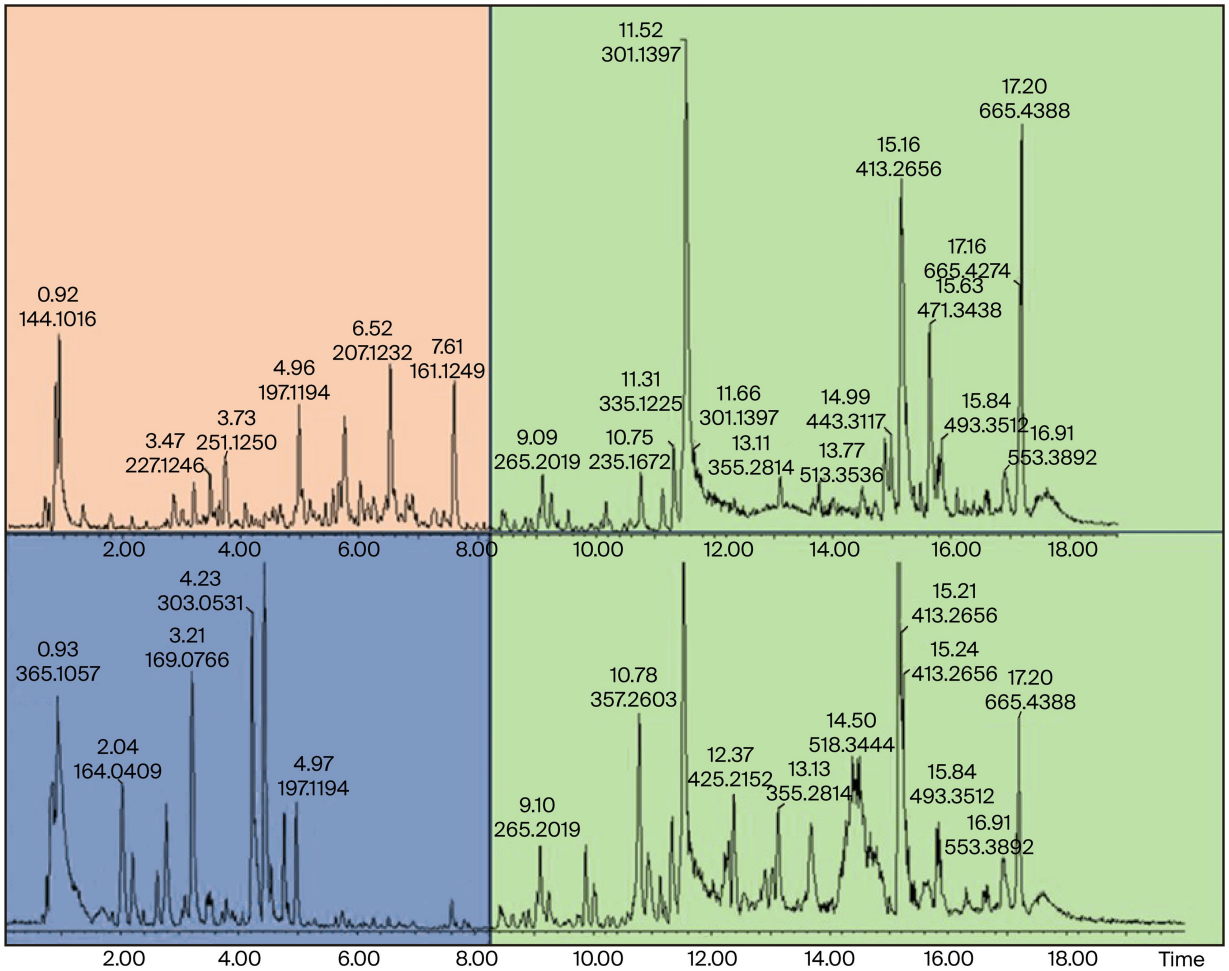
UPLC-ESI-QToF-MS DCM extracts showing phytochemical variation at Rt of 0.92–8.20 min and similarities at 8.25–18.00 min in NGD (top) and SAD (down) *Moringa oleifera* DCM extracts. NG = Nigerian, SA = South African.

Samples 31 and 32 were further analyzed using high-resolution UPLC-ESI-QToF-MS to annotate the marker phytonutrients that these samples contain. The UPLC-MS chromatograms for the methanol and dichloromethane extract are depicted in Figures 6, 7. The overlapping chromatographic peaks, Rt of 8.25–18.00 min in NG (top) and SA (down), suggest the similarities in phytonutrients between the Nigerian and South African *M. oleifera* leaf samples. There were variations in the phytonutrients of the samples—as evident in the South African sample having more phytonutrients (blue box) in this region—than in its Nigerian counterpart (pink; Figure 6) when extracted with methanol. It must be mentioned here that there were many low-abundant metabolites at Rt of 2.2–3.8, 7.8–11.4, and 14.00–15.2 min that were not annotated their respective *m*/*z* ratio automatically and were thus not of interest for further analysis.

The suggested similarities in the phytonutrients of samples from both countries were seen at Rt of 8.25–18.00 min. Two conspicuous phytonutrients with *m*/*z* of 301.1397 and 413.2656 resolved at 11.53 and 15.15 min, respectively. Conversely, the samples extracted with dichloromethane proved that the Nigerian samples have more phytonutrients at Rt 0.92–8.20 min, while the South African samples displayed stark differences in their phytonutrients appearing at Rt 5.0–7.0 min. The primary marker phytonutrients from the dichloromethane extract with *m*/*z* of 301.1397, 413.2666, and 685.4388 resolved at 11.52, 15.16, and 17.20 min, respectively.

The fact that phytonutrients with *m*/*z* = 301.1398 and 413.2656 were extracted by the non-polar dichloromethane and the polar methanol in high abundance of between 85 and 95% suggests these phytonutrients are the potential marker phytonutrients in Nigerian and South African *M. oleifera* leaves.

### 3.4 Structural annotation of marker phytonutrients from Nigerian and South African *Moringa oleifera* leaf methanol extracts

The UPLC-MS analysis of the *M. oleifera* leaf samples in this study was performed using the method reported by Lin and co-workers in 2019 (20) but with minor modifications. The confidence level for this type of annotation may be classified as level 2a because it makes use of MS, MS2, and library MS2 for the annotation of the structures (24). In total, twelve phytonutrients (Table 1) have been identified and proposed as phytonutrients in Nigerian and South African *M. oleifera* leaves. Kaempferol, quercetin, luteolin, tangutorid E, and podophyllotoxin were identified as the significant marker phytonutrients present in *M. oleifera* leaf samples from both countries.

**Table 1:** Phytonutrients identified and proposed as markers in Nigerian and South African *Moringa oleifera* leaves.

Rt (min)	Formula	Accurate mass	Fragmentation pattern from this study	Annotated phytonutrients	Sources	Reference
0.93	C_12_H_22_O_11_	365.1057	85, 90, 127, 145, 162, 163, 281, 325, 362, 365 [M + Na]	Disaccharide	SA	MB
4.23	C_16_H_18_N_2_O_4_	303.0531	89, 135, 179, 303, 304, 305,	Tangutorid E	NG/SA	120
4.77	C_15_H_10_O_6_	287.0566	151, 162, 228, 287 [M + 1]	Kaempferol	NG	19, 20
4.97	C_9_H_8_O_4_	198.1194	107, 133, 134, 135, 180, 198 [M + H_2_0]	*trans*-Caffeic acid	SA/NG	MB
9.10	C_15_H_10_O_6_	287.2019	149, 153, 175, 241, 243, 285, 286.	Luteolin	NG/SA	MB 19
11.31	C_20_H_32_O_4_	335.1225	116, 149, 179, 217, 301, 302, 319, 357.	Prostaglandin A1		MB
11.52	C_15_H_10_O_7_	303.1397	121, 149, 151, 178, 180, 273, 301 302 [M + 1].	Quercetin	NG/SA	MB, 19
13.13	C_16_H_18_O_9_	355.2814	143, 190, 335, 355 [M + 1].	Cryptochlorogenic acid		19, 20
15.16	C_20_H_26_O_9_	413.2656	149, 217, 301, 393, 413, 414, 415.	Podophyllotoxin	NG/SA	MB
15.85	C_20_H_23_N_7_O_7_	493.3512	149, 217, 304, 494, 495, 601, 645, 777	Tiamulin	NG/SA	MB
16.91	C_40_H_54_O	553.3892	124, 149, 217, 261, 275, 551, 552,553 [M + 3H].	Echinenone	NG/SA	MB
17.22	C_15_H_30_O	227.9535	108, 165, 271.	*n*-Pentadecanal	NG/SA	20

MB: https://massbank.eu/MassBank/.

**Figure 8:**
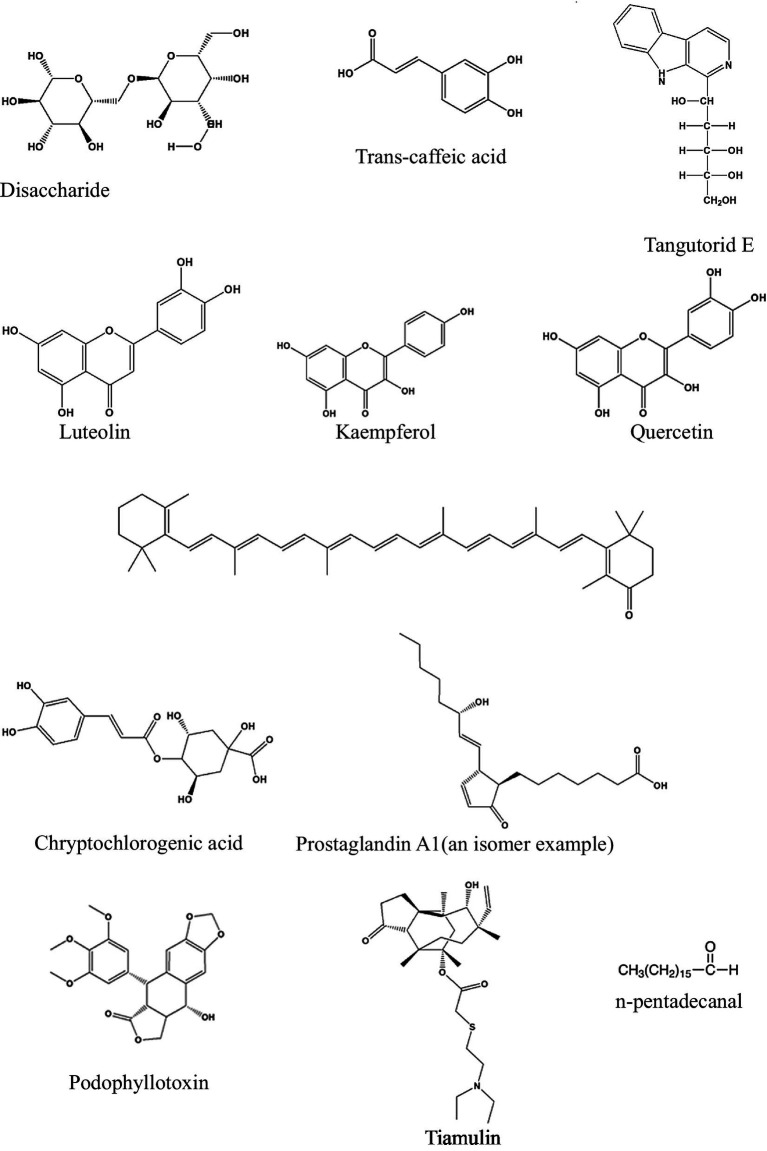
Structures of the phytonutrients identified in Nigeria and South African *Moringa oleifera* leaves.

The structures of the identified phytonutrients are displayed in Figure 8. Considering the different moieties that characterize the structures, the primary marker phytonutrients are polar phenolic phytonutrients; for example, luteolin kaempferol and quercetin.

### 3.5 *In vitro* quantitative antimicrobial activity of the test extracts

The antibacterial activities of all the test extracts—SAM31, SAM32, NGM31, NGM32, SAD31, SAD32, NGD31, NGD32, SADM31, SADM32, NGDM31, and NGDM32 were investigated; the results are listed in Table 2.

**Table 2:** *In vitro* quantitative antimicrobial potentials of the test extracts against selected micro-organisms.

Test extracts	MIC (mg/ml) of microorganisms				
	*Bacillus cereus*	*Escherichia coli*	*Pseudomonas aeruginosa*	*Streptococcus pyrogenes*	*Staphylococcus aureus*
MeOH*	1.25	1.25	1.25	1.25	1.25
DCM*	1.25	1.25	1.25	1.25	1.25
MeOH-DC (1:1 v/v)*	1.25	1.25	1.25	1.25	1.25
Ciprofloxacin	0.0039	0.0156	0.0078	0.0039	0.0039

*Extracts from Nigeria and South African *Moringa oleifera* Lam. leaves.

#### 3.5.1 *In vitro* quantitative assessment of antioxidant activity of test extracts by DPPH assay

**Figure 9:**
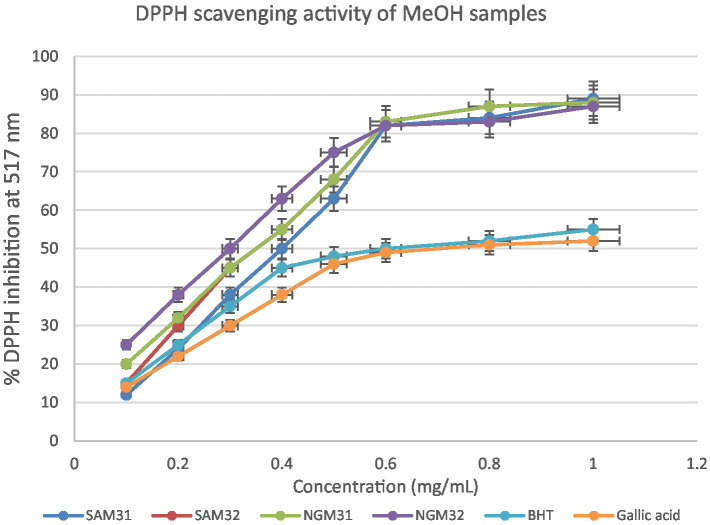
2,2-Diphenyl-1-picrylhydrazyl (DPPH) scavenging activity of methanol extracts from South Africa and Nigeria including the standards Gallic acid and butylated hydroxyl toluene (BHT). Each value is expressed as mean ± standard deviation of (*n* = 3).

**Figure 10:**
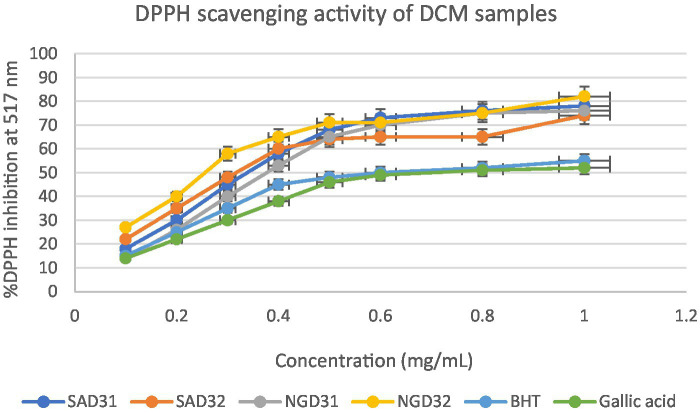
2,2-Diphenyl-1-picrylhydrazyl (DPPH) scavenging activity of dichloromethane (DCM) extracts from South Africa and Nigeria including the standards gallic acid and butylated hydroxyl toluene (BHT). Each value is expressed as mean ± standard deviation of (*n* = 3).

**Figure 11:**
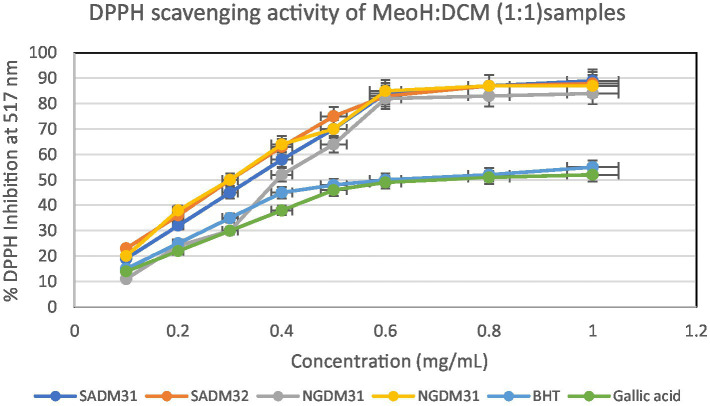
2,2-Diphenyl-1-picrylhydrazyl (DPPH) scavenging activity of 1:1 extracts from South Africa and Nigeria including the standards gallic acid and butylated hydroxyl toluene (BHT). Each value is expressed as mean ± standard deviation of (*n* = 3).

This section delves into the quantitative evaluation of the antioxidant activity of *M. oleifera* extracts, utilizing the DPPH free radical scavenging assay. The assay outcome yielded positive antioxidant potentials for all the test extracts (Figures 9–11), and the IC_50_ values for the extracts and standards are listed in Table 3. The antioxidant assays unveiled concentration-dependent trends with significant DPPH assays scavenging activity of up to 98%, with IC_50_ = 0.14 mg/mL.

**Table 3:** 2,2-Diphenyl-1-picrylhydrazyl (DPPH) and H_2_O_2_ free radical scavenging (antioxidant) potentials minimum inhibitory concentration (MIC) half-maximal inhibitory concentration (IC_50_) values (mg/ml) of Nigerian and South African *Moringa oleifera* Lam. leaves vs. positive control extracts.

	IC_50_ (mg/ml)	
Extracts	H_2_O_2_	DPPH
SAM31	0.42	0.41
SAM32	0.38	0.40
NGM31	0.36	0.35
NGM32	0.30	0.14
SAD31	0.40	0.33
SAD32	0.41	0.19
NGD31	0.45	0.38
NGD32	0.28	0.34
SADM31	0.35	0.37
SADM32	0.31	0.28
NGDM31	0.44	0.24
NGDM32	0.31	0.25
BHT	0.71	0.66
Gallic acid	0.77	0.57

#### 3.5.2 *In vitro* quantitative assessment of antioxidant activity of test extracts by hydrogen peroxide (H_2_O_2_) assays

**Figure 12:**
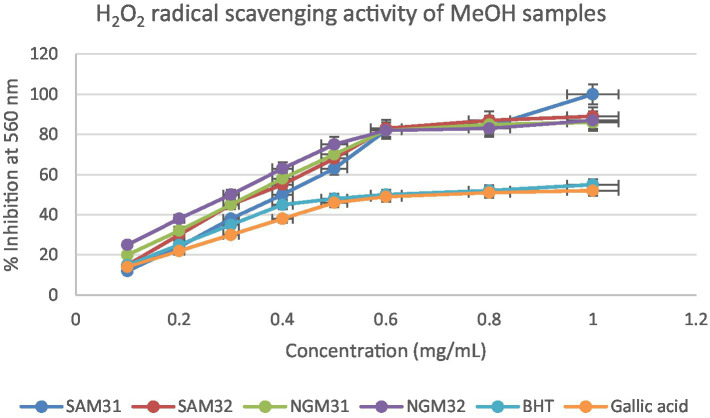
Hydrogen peroxide radical scavenging activity of methanol extracts from South Africa and Nigeria including the standards gallic acid and butylated hydroxyl toluene (BHT). Each value is expressed as mean ± standard deviation of (*n* = 3).

**Figure 13:**
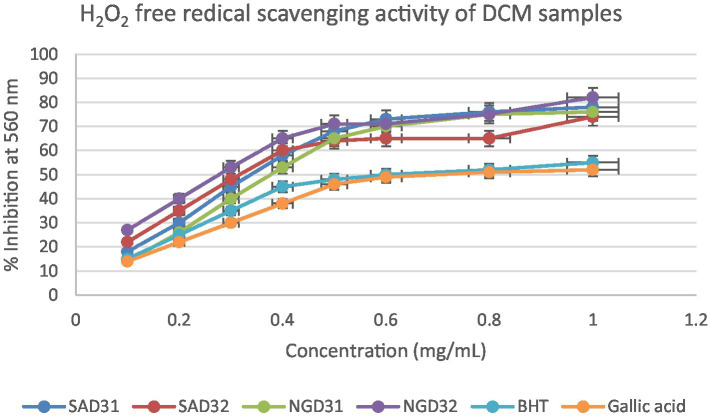
Hydrogen peroxide radical scavenging activity of dichloromethane (DCM) extracts from South Africa and Nigeria including the standards gallic acid and butylated hydroxyl toluene (BHT). Each value is expressed as mean ± standard deviation of (*n* = 3).

**Figure 14:**
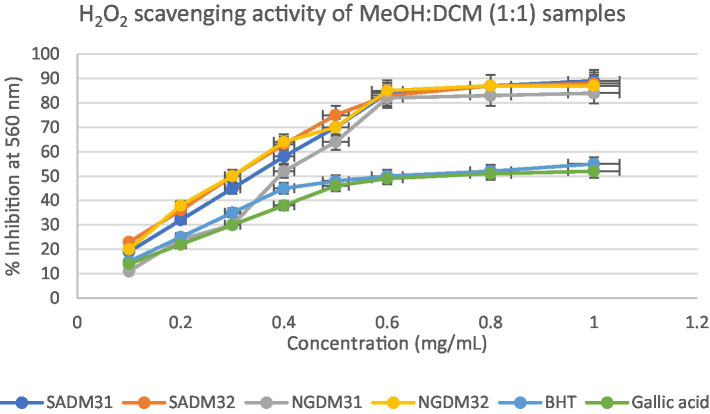
Hydrogen peroxide radical scavenging activity of dichloromethane (DCM) extracts from South Africa and Nigeria including the gallic acid and butylated hydroxyl toluene (BHT) standards. Each value is expressed as mean ± standard deviation of (*n* = 3).

The investigation into the antioxidant capacity of *M. oleifera* extracts was repeated using a hydrogen peroxide assay. The graphical visualization of the obtained results is depicted in Figures 12–14. The hydrogen peroxide assay demonstrated consistent inhibitory percentages but with relatively lower free radical scavenging potentials of 87% with IC_50_ = 0.28 mg/mL.

It is evident in the Figures that all the extracts also displayed concentration-dependent free radical scavenging activity, with a 10–70% inhibitory activity at a concentration range of 0.1–0.5 mg/mL. However, from a concentration of 0.5 mg/mL, the extracts methanol, dichloromethane, and their 1:1 v/v combination tend to perform better with inhibitory activity between 75–90% for the extracts in comparison to 50–60% for the standards gallic acid and BHT positive controls used in the study.

Overall, all the test extracts exhibited dose-dependent better antioxidant potentials than standard BHT and gallic acids used as positive controls, thus highlighting the antioxidant health benefits of commercial products formulated from Nigerian and South African *M. oleifera* Lam. leaves.

### 3.6 Half-maximal inhibitory concentration (IC_50_) values of test extracts used in DPPH and H_2_O_2_ assays

The half-maximal inhibitory concentration (IC_50_) is a crucial parameter that quantifies the concentration of an extract required to inhibit 50% of the free radicals or hydrogen peroxide activity. The IC_50_ values for each extract were calculated from the concentration–response curves generated in both assays, and the results are displayed in Table 3.

## 4 Discussion

### 4.1 OPLS-DA analysis of HPLC-PDA data of Nigerian and South African *Moringa oleifera* extracts

According to the OPLS-DA and the HCA analysis of Nigerian and South African *M. oleifera* leaf methanol extracts, many samples from both countries exhibit a significant similarity in their phytonutrients. At the same time, a few expressed the apparent discrepancies in the phytochemicals of the samples. The above-average trend in the similarities and dissimilarity in the phytoconstituent in the *M. oleifera* leaves has been reported for samples collected from India and China (20). According to the authors, about 96% of the samples investigated expressed similarities in their phytonutrients, that is, 118 out of the 122 samples indicated similarity and a shared phytochemical component. From this study, the HCA plot depicted in Figure 3 and the representative chromatograms shown in Figures 5, 6 depict the area of similarity in green color and the area with differences in blue and brown.

### 4.2 Marker samples nomination, profiling, and structural annotation from Nigerian and South African *Moringa oleifera* extracts by OPLS-DA analysis of HPLC-PDA data

From the marker samples annotated by OPLS-DA and their structural annotations (Figure 8), 12 phytonutrients were identified from the Nigerian and South African *M. oleifera* extracts. From that number, there are three phenolics phytonutrients: luteolin, kaempferol, and quercetin; one alkaloid, tiamulin; a phenolic acid, cryptochlorogenic acid; and four lactones, among others. This supports the above-average similarities in the phytonutrients of Nigerian and South African *M. oleifera* leaves. The aforementioned similarities, however, do not encourage the interchangeable use of *M. oleifera* leaf samples from both countries, as this could result in dire consequences, including death. This is because of the several unidentified peaks present in the chromatogram, as shown in Figures 6, 7; so, whether these phytonutrients, when eventually identified, would be toxic or not does not speak in favor of the interchangeable use of samples from both countries. A plethora of on-the-go herbal immune system boosters, antidiabetic decoctions, general detoxifying agents, so-called sex activity boosters or manpower drinks in different formulations floods the South African low to medium market from Nigeria and vice versa, considering these two countries are the most significant economic hubs in sub-Saharan Africa. The majority of the product labels generally tell the user the products contain herbal or medicinal plant extracts without scientific validation of the specific plant that was extracted. However, results obtained from this study recommend that luteolin, kaempferol, quercetin, and cryptochlorogenic acid, found in both the polar and non-polar extracts of Nigerian and South African *M. oleifera* leaves among other phytonutrients may serve as benchmark standard phytonutrients that may be utilized for the quality control of the plethora of on-the-go herbal products that influx the Nigerian and South African markets.

### 4.3 *In vitro* quantitative antimicrobial activity of the test extracts

According to Gibbons (25), 4 μg/mL MIC for a natural product would suitably mitigate pathogenic effects on host cells. Similar to previous reports (26, 27), all the test extracts did not exhibit a good-to-significant antibacterial inhibitory effect (MIC = 1.25 mg/mL) compared to ciprofloxacin (MIC = 0.0156–0.0039 mg/mL). However, the antimicrobial potentials of the marker samples from this study show that there is little antimicrobial activity at the selected concentration of the different samples; these findings are in line with certain studies from the literature (26, 27). Accordingly Onsare et al. (28), documented less efficiency of the extracts of *M. oleifera* on *P. aeruginosa*, while Kusuma et al. (29) and Rahman et al. (30) also reported less activity to eradicate the growth of *Bacillus cereus* and other test bacteria at significant MIC values (< 1 mg/mL). On the contrary, a review by Nielsen et al. (31) suggests that medicinal extracts with 8,000 μg/mL possess antimicrobial activity, although this is not considered significant (<1 mg/mL).

### 4.4 *In vitro* free radical scavenging evaluations of the *Moringa* extracts

#### 4.4.1 *In vitro* quantitative assessment of antioxidant activity of test extracts by DPPH assay

The investigation into the antioxidant activity of *M. oleifera* extracts involved two distinct assays: the DPPH and hydrogen peroxide (H_2_O_2_) assays. These assays provide insights into the extracts’ radical scavenging and hydrogen peroxide inhibitory capacities.

The DPPH assay is a widely employed method to qualitatively assess the ability of substances to act as radical scavengers or antioxidants. The results of the DPPH assay for *M. oleifera* extracts are shown in the provided chart (Figures 6–11). The percentages represent the degree of discoloration or absorbance decrease, indicative of the antioxidant potential of each extract at different concentrations.

SAM31 and SAM32 exhibited a significant increase in antioxidant activity with higher concentrations, reaching up to 98% scavenging at the highest concentration. NGM32 demonstrated higher antioxidant activity, achieving 98% scavenging at the highest concentration compared to NGM31. Antioxidant activity for SAD32 increases with concentration, reaching 91% scavenging at the highest concentration. NGD32 demonstrated higher scavenging percentages, peaking at 99% at the highest concentration. SADM32 and NGDM32 extracts display substantial antioxidant potential, with SADM32 reaching 97% scavenging at the highest concentration. NGDM32 reaches 98% scavenging at the highest concentration. BHT and gallic acid, serving as standards, show a dose-dependent increase in antioxidant activity. The hydrogen peroxide assay evaluated the extracts’ ability to inhibit hydrogen peroxide activity, providing insights into their antioxidant potential, and the table presents inhibition percentages at different concentrations.

#### 4.4.2 *In vitro* quantitative assessment of antioxidant activity of test extracts by hydrogen peroxide (H_2_O_2_) assays

The hydrogen peroxide assay evaluated the extracts’ ability to inhibit hydrogen peroxide activity, providing insights into their antioxidant potential. The results, presented in the provided chart (Figures 12–14), indicated the inhibition percentages at different concentrations. Both samples of SAM31 and SAM32 consistently demonstrated increased hydrogen peroxide inhibition, reaching up to 89 and 88%, respectively. In the case of NGM31 and NGM32, NGM32 exhibited higher inhibition percentages, reaching 87% at the highest concentration. SAD32, among the SAD31 and SAD32 samples, demonstrated inhibition up to 78% at the highest concentration. In the NGD31 and NGD32 samples, NGD32 displayed higher inhibition percentages, peaking at 82% at the highest concentration. For SADM31 and SADM32, SADM32 exhibited increased inhibition, reaching 89% at the highest concentration. NGDM31 and NGDM32 samples show varying degrees of inhibition, with NGDM32 reaching 87% at the highest concentration. Previous studies have reported similar antioxidant potentials of *M. oleifera* samples from Nigeria (32) and South Africa (33). The DPPH and hydrogen peroxide assays demonstrate that *M. oleifera* extracts possess substantial antioxidant potential. The variations in antioxidant activity among samples may be attributed to differences in phytochemical composition influenced by geographical factors. The concentration-dependent trends in both assays emphasized the importance of extract concentration in achieving optimal antioxidant efficacy. In addition, the overall higher antioxidant potential of the methanol extracts over the dichloromethane, and the methanol-dichloromethane (1:1 v/v) extracts serves as a function of solvent polarity. That is, methanol with better polarity over dichloromethane and methanol-dichloromethane (1:1 v/v) solubilized more polyphenolic compounds for better antioxidant performance.

### 4.5 Half-maximal inhibitory concentration (IC_50_) values of test extracts used for DPPH and H_2_O_2_ assays

The IC_50_ values, which quantitatively measured the antioxidant potency of each extract, afforded values ranging from 0.14 mg/mL for Nigerian methanol extracts (NGM32) to 0.41 mg/mL for South African methanol extract (SAM31) from the DPPH assay. At the same time, the H_2_O_2_ free radical assay revealed 0.28 mg/mL for the NGM32 compared to 0.42 mg/mL for the SAM32 extracts. With a single factor ANOVA *p*-value of 0.17, the variance within and between the group of samples and the two antioxidant assays was considered statistically insignificant about the geographic locations where the *M. oleifera* leaf samples were harvested. The better performance of the methanol extract over the dichloromethane extract was expected as the methanol extract should be more prosperous in polyphenols characterized by antioxidant activities. However, what came as a surprise to us was that all the extracts out-performed the BHT and gallic acid that exhibited IC_50_ values of 0.66–0.57 mg/mL for the DPPH assay and 0.71–0.77 mg/mL for the H_2_O_2_ assay, respectively. Our results, including IC_50_ values, affirm the antioxidant prowess of *M. oleifera* extracts, positioning them as promising sources of natural antioxidants. This study contributes valuable insights into the potential health benefits of these extracts, laying the foundation for further exploration of their applications in medicine and healthcare.

## 5 Conclusion

To consider the safe use of *M. oleifera* plant extracts, evaluating its cytotoxicity *in vitro* and *in vivo* is recommended since toxicity depends on the phytonutrients. Whereas the use of edible solvents such as water should be considered for extraction in the future studies to investigate food-derived phytonutrients, this study is the first of its type for African *M. oleifera* leaves as far as our knowledge is concerned. Therefore, it serves as a benchmark toward explaining the phytonutrients and marketing implications on the quality of products formulated with samples harvested from different growth environments in Africa, especially South Africa and Nigeria.

## Data Availability

The original contributions presented in the study are included in the article/supplementary material, further inquiries can be directed to the corresponding author.
